# Distinct Correlation Structure Supporting a Rate-Code for Sound Localization in the Owl’s Auditory Forebrain

**DOI:** 10.1523/ENEURO.0144-17.2017

**Published:** 2017-06-30

**Authors:** Michael V. Beckert, Rodrigo Pavão, José L. Peña

**Affiliations:** Dominick P. Purpura Department of Neuroscience, Albert Einstein College of Medicine, Bronx, NY 10461

## Abstract

While a topographic map of auditory space exists in the vertebrate midbrain, it is absent in the forebrain. Yet, both brain regions are implicated in sound localization. The heterogeneous spatial tuning of adjacent sites in the forebrain compared to the midbrain reflects different underlying circuitries, which is expected to affect the correlation structure, i.e., signal (similarity of tuning) and noise (trial-by-trial variability) correlations. Recent studies have drawn attention to the impact of response correlations on the information readout from a neural population. We thus analyzed the correlation structure in midbrain and forebrain regions of the barn owl’s auditory system. Tetrodes were used to record in the midbrain and two forebrain regions, Field L and the downstream auditory arcopallium (AAr), in anesthetized owls. Nearby neurons in the midbrain showed high signal and noise correlations (R*_NC_*s), consistent with shared inputs. As previously reported, Field L was arranged in random clusters of similarly tuned neurons. Interestingly, AAr neurons displayed homogeneous monotonic azimuth tuning, while response variability of nearby neurons was significantly less correlated than the midbrain. Using a decoding approach, we demonstrate that low R*_NC_* in AAr restricts the potentially detrimental effect it can have on information, assuming a rate code proposed for mammalian sound localization. This study harnesses the power of correlation structure analysis to investigate the coding of auditory space. Our findings demonstrate distinct correlation structures in the auditory midbrain and forebrain, which would be beneficial for a rate-code framework for sound localization in the nontopographic forebrain representation of auditory space.

## Significance Statement

Despite their established involvement in sound localization, our understanding of how the midbrain and forebrain encode sound location is limited. An outstanding difference between these regions is the lack of obvious topographic representations of auditory space in the forebrain. To shed light on the circuit function, we examined the tuning and correlation structure in responses of nearby neurons in the midbrain and forebrain. Interestingly, a different correlation structure emerged in the forebrain: uniform tuning shape and uncorrelated response variability. This finding highlights differences between the midbrain and forebrain representation of auditory space and provides evidence supporting a rate code for sound location in the forebrain.

## Introduction

**Figure 1.**
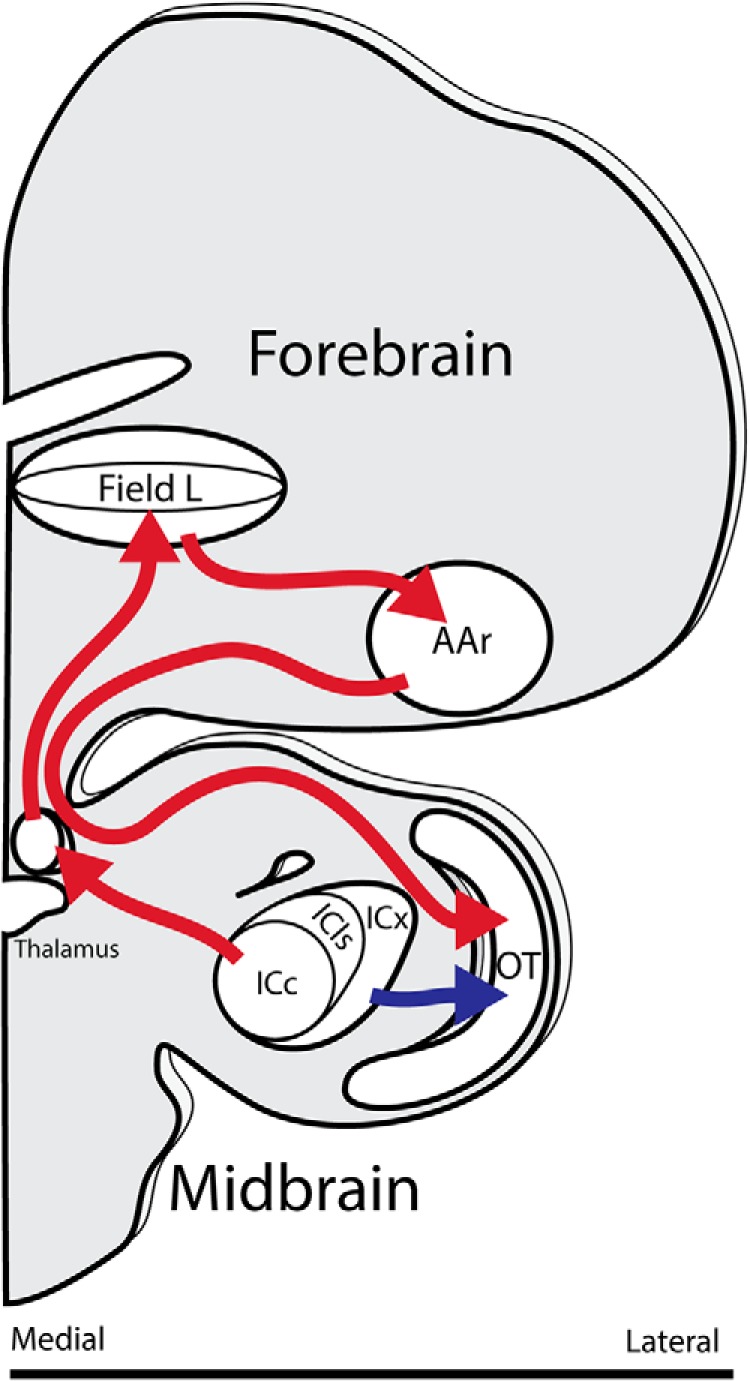
Schematic of tectal (blue) and forebrain (red) auditory pathways of the owl’s brain. The auditory midbrain consists of subdivisions of the inferior colliculus: the central core (ICc), lateral shell (ICls), and external nucleus (ICx). The map of auditory space first emerges in ICx. ICx projects to the OT, analog to the superior colliculus. The forebrain pathway originates in projections from the inferior colliculus to the thalamus. The auditory forebrain structure Field L, analog to primary auditory cortex, displays a clustered nontopographic tuning to binaural cues. Field L projects directly to the AAr, analog to the auditory portion of the frontal eye fields. AAr sends projections back onto OT. For clarity, some connections are omitted.

The barn owl is a nocturnal predator capable of hunting in darkness using auditory cues (Payne, 1971). Owls can use interaural time difference (ITD; Moiseff and Konishi, 1981) across their entire hearing range, 100–10,000 Hz (Köppl, 1997), to calculate the horizontal position (azimuth) of sound sources with high acuity (Knudsen et al., 1979; Moiseff, 1989; Poganiatz et al., 2001). Downstream from the brainstem, the owl’s auditory system separates into the tectal and forebrain pathways (Fig. 1). Head-orienting behavior to sound is maintained or recovers after lesion of the forebrain or tectal pathways, respectively, but is lost when both are ablated, suggesting one pathway may compensate for the loss of the other and either is sufficient to support the function (Knudsen et al., 1993; Wagner, 1993). Yet, the spatial tuning of neurons is arranged differently in these pathways. The tectal pathway displays a map of auditory space in the external nucleus of the inferior colliculus (Knudsen and Konishi, 1978) and the optic tectum (OT; Knudsen and Knudsen, 1983), analogous to the superior colliculus (Knudsen, 1982). In contrast, the forebrain regions the auditory arcopallium (AAr), analogous to the auditory portion of cortical frontal eye fields (Knudsen et al., 1995), and its primary input region, Field L, analogous to primary auditory cortex (Cohen et al., 1998), contain random clusters of similarly tuned neurons (Cohen and Knudsen, 1995, 1998). This nontopographic organization is also observed in the mammalian auditory cortex, e.g., nonhuman primates (Benson et al., 1981), cats (Eisenman, 1974; Middlebrooks and Pettigrew, 1981), and bats (Razak et al., 2015). The qualitatively distinct organization of midbrain and forebrain reflects differences in network architecture and suggests that coding schemes across brain regions may also differ.

An essential difference between topographic and nontopographic representations is the relationship between the tuning of neighboring cells. Specifically, the tuning of nearby cells in a topographic representation is similar and predictable. From a connectivity viewpoint, similarity of tuning in nearby cells reflects shared inputs. The amount of shared inputs by nearby cells also determines their trial-by-trial response variability (Shadlen and Newsome, 1998). Tuning similarity and trial-by-trial variability are known as signal (Gawne and Richmond, 1993) and noise (van Kan et al., 1985) correlation, respectively, and jointly referred to as the correlation structure (Zohary et al., 1994; Bair et al., 2001; Seriès et al., 2004; Averbeck et al., 2006). An additional indicator of shared inputs is spike synchrony (Miller et al., 2014; Atencio et al., 2016; Sabri et al., 2016; Schwab et al., 2017; Yaeger and Trussell, 2016).

The impact of correlations depends on many factors: coding scheme (Paradiso, 1988; Seung and Sompolinsky, 1993; Butts and Goldman, 2006; Latham and Roudi, 2013), network architecture, and upstream computations (Kohn et al., 2016). The extensive description of sound localization mechanisms in the owl’s brain (Takahashi, 2010; Konishi, 2012; Wagner et al., 2013) makes this system well suited for insightful interpretations. Specifically, we considered the two-channel theory of how ITD is encoded for sound localization. This theory postulates that differential activity in two hemispheric populations can explain lateralization of sound sources (Békésy, 1930; van Bergeijk, 1962). There is growing evidence that this coding scheme is implemented in rodents (McAlpine et al., 2001; Grothe et al., 2010) and humans (Briley et al., 2013; Derey et al., 2016; McLaughlin et al., 2016). The competing hemispheric channels would result from populations of neurons with mirrored monotonic tuning. Because noise correlation (R*_NC_*) affects information drawn from the firing rate of homogeneously tuned populations (Ecker et al., 2011; Zohar et al., 2013), it could impact information under this coding scheme.

Correlation analysis requires simultaneous recording of multiple neurons, a task which has been notoriously difficult in the auditory system (Syka et al., 1981; Gray et al., 1995; Richardson et al., 2013). To this end, we conducted tetrode recordings for the first time in the owl’s auditory regions OT, Field L, and AAr. Nearby OT cells were similarly tuned and their responses covaried, consistent with a topographic representation. On the other hand, Field L showed clusters of similarly tuned neurons, consistent with previous reports (Cohen and Knudsen, 1998). Intriguingly, cells in AAr displayed uniform tuning across the population and strikingly low R*_NC_* and synchrony of nearby cells. Furthermore, we show AAr’s correlation structure is beneficial under a rate-code framework. These findings demonstrate that the difference between the midbrain and forebrain goes beyond large scale topography, displaying distinct correlation structures that may be important for how sound location is encoded in the forebrain.

## Materials and Methods

### Subjects and surgery

Adult American barn owls (*Tyto furcata*) of both sexes (four male and one female) were implanted with custom built stainless steel headplates (Einstein Engineering Department). Dental acrylic was used to form a molded well aimed over OT, Field L, and AAr for repeated recordings.

Owls were food deprived 12 h preceding each recording session. During recording sessions, owls were anesthetized with intramuscular injections of ketamine (Ketaset; 20 mg/kg) and xylazine (Anased; 2 mg/kg), along with prophylactic antibiotics (ampicillin; 20 mg/kg, i.m.) and lactated Ringer’s solution (10 ml, s.c.). Anesthesia level was assessed by pedal and eyelid reflex. Subsequent half doses of ketamine and xylazine were administered throughout the recording session as needed, to maintain a proper anesthesia level. Body temperature was maintained with a heating pad.

At the end of each session the craniotomy and well in the head cap was sealed with a silicone compound (Quick-Pro, Warner Tech-Care). An analgesic was administered intramuscularly (3 mg/kg; Rimadyl) to prevent inflammation and pain. Owls were allowed to recover overnight in a small crate. They were returned to the home aviary when all physical impairment signs were absent. Owls recovered for >10 d before another recording session. All procedures were in compliance with guidelines set by the National Institutes of Health and Albert Einstein College of Medicine’s Institute for Animal Studies.

### Data collection

All recordings were performed in a double wall sound attenuated chamber (Industrial Acoustics), lined with anechoic acoustic foam (Sonex). OT, Field L, and AAr were targeted stereotaxically using known coordinates relative to the intersection of the midline and interaural line as well as by established physiologic response properties: response latency, spontaneous firing rate, and tuning to ITD, interaural level difference (ILD), and frequency (Knudsen and Konishi, 1978; Knudsen and Knudsen, 1983; Knudsen et al., 1993, 1995; Wagner, 1993; Cohen and Knudsen, 1995, 1996, 1998; Cohen et al., 1998; Vonderschen and Wagner, 2009, 2012).

Tetrodes (Q-trodes, NeuroNexus) were advanced through small openings in the dura made with a sterile needle, using a micromanipulator (David Kopf Instruments). Tetrodes were chosen to achieve simultaneous recording of multiple nearby single units which is necessary to perform correlation analysis. Data acquisition was performed using a Plexon Omniplex system (SortClient, Plexon). We recorded sites containing at least two visually well-isolated units. After the recording, isolation was confirmed with offline sorting software (Offline Sorter, Plexon), with an average of four to five units separated per site.

Collecting the data necessary for the analysis of signal and R*_NC_*s and synchrony, under dichotic and free-field stimulation required long acquisition times for each recording site. Thus, often recordings were not successfully held for the amount of time necessary to obtain data for every type of analysis. The sample size of each dataset is provided in the results.

### Acoustic stimulation

#### Dichotic stimulation

Dichotic (earphone) stimulation was used to identify recording sites. Acoustic stimulation was performed using previously described methods (Steinberg and Peña, 2011; Wang et al., 2012; Steinberg et al., 2013; Wang and Peña, 2013; Cazettes et al., 2014, 2016). Briefly, Tucker-Davis Technologies System 3 and custom written MatLab (Mathworks) routines were used to synthesize and deliver all acoustic stimuli. Custom-made earphones (Einstein Engineering Department) containing a speaker (Knowles, model 1914) and a microphone (Knowles, model 1319) were inserted into the owl’s ear canal. The earphone microphones were then used to correct irregularities in phase and level across frequency of earphone speakers each time they were positioned in the ear canals. The tuning to ITD and ILD as well as frequency was used to identify recording sites by the response properties characteristic of OT, Field L, and AAr neurons.

After a site was confirmed with dichotic stimulation, the earphones were removed to allow for free-field stimulation.

#### Free-field stimulation

Free-field sound stimulation was presented through a custom built spherical array of speakers (Sennheiser, 3P127A) surrounding the stereotax (Pérez and Peña, 2006; Pérez et al., 2009; Wang et al., 2012; Wang and Peña, 2013). Speaker positions ranged from ±100° azimuth and ±80° elevation with spacing between 10° and 30°. For combining data across hemispheres, azimuth was normalized such that positive values corresponded to contralateral space relative to the recording side. Owls were positioned to face the 0° azimuth and 0° elevation speaker for all recordings. Speakers were calibrated using a Brüel and Kjær microphone (model 4190). Broadband signals (500 Hz to 10 kHz) were transformed by the calibration filter for each speaker to equalize sounds across the array. Stimulus duration and interstimulus intervals were the same as those used for site confirmation with dichotic stimulation (150 and 300 ms, respectively). Speakers were activated randomly 20–40 times to measure a spatial receptive field (SpRF).

After the free-field stimulation protocol was completed, the earphones were repositioned and recalibrated, to search for subsequent recording sites.

### Data analysis

#### Tuning curves

**Figure 2.**
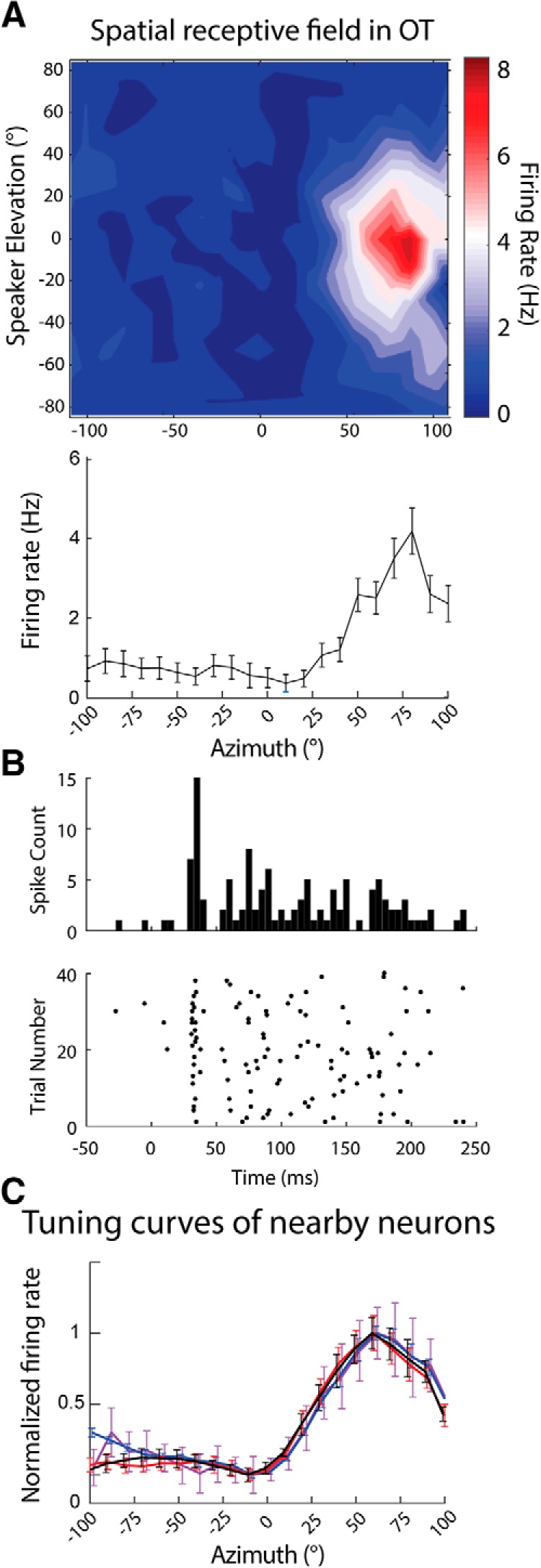
Azimuth tuning of nearby neurons in OT. ***A***, Spatial receptive field (SpRF) (top) and azimuth tuning curve (bottom) obtained by averaging the SpRF across elevation. ***B***, Peristimulus time histogram (PSTH; top) and raster (bottom) for the spiking activity of the neuron in ***A*** responding to sound at the preferred direction (90° azimuth and 0° elevation). ***C***, Example azimuth tuning curves of neurons recorded from the same site. Responses are normalized to facilitate visual comparison. Tuning curves represent mean ± SEM, 20–40 repetitions.

Action potentials occurring during the stimulus (150-ms window after stimulus onset) were binned and averaged to generate SpRFs. The mean firing rate within a window equivalent to the stimulus duration that preceded the stimulus onset was used to assess spontaneous activity. Neurons were included in the sample if they showed a significant response to sound, i.e., if the firing rate during sound stimulation was two standard deviations above the spontaneous activity, and considered tuned if the mean peak activity of the tuning curve was two standard deviations above the lowest mean response. SpRFs were transformed into azimuth tuning curves by averaging response across speakers with equivalent azimuths (Fig. 2*B*). Azimuth tuning curves for Field L and AAr were smoothed using 30° sliding windows. This method has been used to facilitate the characterization of tuning curves in Field L and AAr (Vonderschen and Wagner, 2009). The same procedure was used to generate ITD tuning curves in Field L and AAr to assess the similarity of shape across recording sites.

To further characterize AAr tuning, azimuth curves were subdivided into three regions: frontal (±40°), contralateral (+50° to +100°) and ipsilateral (−50° to −100°) portions. The slope of the tuning curves within each of these regions was assessed by computing the mean slope of linear regressions for a sliding window of three consecutive curve data points, spanning over 30° in azimuth. This method permitted a fine description of the change in slope while smoothing out noise.

### Correlation analysis

Tuning similarity was assessed with the commonly used signal correlation (Bair et al., 2001; Sompolinsky et al., 2001; Kohn and Smith, 2005; Lyamzin et al., 2010; Chelaru and Dragoi, 2016). Signal correlation is the Pearson product-moment correlation coefficient (*R*) for tuning curves of pair of neurons (Liu et al., 2013). Correlation coefficients (R*_sig_*) were converted using Fisher’s z-transformation for statistical purposes and converted back to *R* values for reporting (Silver and Dunlap, 1987; Kohn and Smith, 2005; Smith and Kohn, 2008). Signal correlations in pairs of simultaneously recorded neurons are referred to as correlations computed “within” recording sites. Additionally, for Field L and AAr, signal correlations were computed “across” recording sites (not simultaneously recorded) to examine the homogeneity of tuning across the population.

R*_NC_* is the trial-by-trial response variability of pairs of neurons over repeated presentations of a frozen (identical) stimulus. This is the Pearson correlation coefficient of spike counts per trial (150 ms each trial; Bair et al., 2001; Kohn and Smith, 2005; Smith and Kohn, 2008; Liu et al., 2013). The calculation of a correlation coefficient is affected by the sample size, i.e., the number of trials, used for each pair. Consider a model distribution of spike counts with a known correlation. As sample size increases the calculated correlation will on average approach the known value; while with few samples the variability of calculated correlations increases. Thus, a single measurement of correlation with a small sample size may not accurately represent the true correlation of a pair. However, the average of repeated measures would be closer to the actual value and could be used to better estimate the correlation (Schönbrodt and Perugini, 2013). To ensure the accurate assessment of the strength of R*_NC_*s, R*_NC_* was calculated for each free-field speaker and then averaged. If a neuron is quiescent, this can also yield a similar effect to reducing the sample size. Therefore, only speakers that elicited a response in at least three trials were included. R*_NC_* was converted using Fisher’s z-transformation for statistics and comparisons. Z values were then converted back to *R* values for reporting.

The synchrony of two simultaneously recorded neurons was computed by generating cross-correlograms (CCGs) of their spike trains (Bair et al., 2001; de la Rocha et al., 2007; Smith and Kohn, 2008). Continuous neural data were converted into binary sequences representing the presence of spikes in time (Offline Sorter, Plexon). CCGs with lags of ±100 ms within a 1-ms bin size were computed for either the duration of the stimulus (150 ms) or an equivalent amount of time preceding the stimulus onset, evoked and spontaneous, respectively. CCGs were then smoothed using a 5-ms sliding window and normalized by the geometric mean firing rate of the neurons and size of the analysis window (Bair et al., 2001). The magnitude of the CCG will increase with firing rate as spikes coincide due to chance alone. To correct for this, a shifted CCG was computed and smoothed. In this case the spike train of one neuron for one trial (n*_i_*) was compared with the spike train of the other neuron on the subsequent trial (n*_i+1_*). This shifted CCG was subtracted from the original CCG to compute the corrected CCG. Synchrony was then quantified by the integral of the peak of the CCG at 0-lag. The peak boundaries were selected by a half-maximum algorithm. For each pair, average synchrony values were obtained from all trials using unfrozen noise stimulation that evoked a significant response (see above, Tuning curves) during free-field and dichotic stimulation.

### Decoding analysis

Azimuthal information in the firing rate of OT, Field L, and AAr neurons was assessed using linear discriminant analysis (Fisher, 1936; Quiroga et al., 2007). The decoder was trained with pairs of simultaneously recorded neurons. This allowed us to train the decoder with actual trial-by-trial neural responses and preserved the embedded correlation structure. This procedure permits a parsimonious estimate of the information contained in a neural population compared with bigger pools which include nonsimultaneously recorded neurons (Miller and Recanzone, 2009; Day and Delgutte, 2013; Goodman et al., 2013; Belliveau et al., 2014). This particular decoder (built-in MatLab function “classify,” Mathworks) determines the linear boundary between measures that maximally separates the responses to different classes of stimuli, in this case the firing rates of pairs of neurons that separate azimuths. This boundary depends on the signal and R*_NC_*s for the pair (Averbeck et al., 2006). For example, let us consider two neurons responding to two sounds from different speakers in azimuth 1 and 2. If the tunings of these neurons are positively correlated, such that both respond strongly to 1 and weakly to 2 and the variability of their responses is independent (low R*_NC_*s), then the optimal classifier would form a line orthogonal to the identity line (i.e., where the responses of the two cells are perfectly matched) lying between the responses of these neurons to each stimulus trial plotted against one another. Alternatively, if the tunings for the neurons are inversely correlated, then the discrimination line that maximally separates responses would be the identity line.

The decoder was trained with neural responses to repeated trials across all speaker positions labeled by their azimuths, to determine the optimal discrimination lines that correctly classified these categories. To exclude noninformative portions of the tuning curves from the analysis of decoding performance, the 21 azimuth categories (±100° in steps of 10°) were down-sampled to 7 by merging three consecutive azimuths. This bin size preserved the shape of each tuning curve while eliminating redundancy. Signal correlations computed for original and down-sampled curves were strongly correlated for all regions [correlation coefficients (*R*): OT = 0.94, Field L = 0.91, AAr = 0.91; *p* < 0.0001 for all]. The decoder was cross-validated by the “leave one out” protocol, i.e., the procedure was repeatedly run where each iteration used the responses from one trial for testing and all remaining trials for training, until each trial had been used for testing once. The decoder’s accuracy (percentage of correct responses) was used as a metric for performance. To assess significance, accuracy was compared with chance level (14.29%, since there are seven categories).

To investigate the effect of signal and R*_NC_*s on the decoder’s performance, we fit linear regressions to the distributions of signal and R*_NC_*s of each pair plotted against decoding accuracy. This tested how predictive the noise and signal correlations were of performance. We calculated the coefficient of determination (*R* ^2^) to quantify the fraction of explained variance described by the regression. We used the built-in MatLab function “regress” (Mathworks) for parameter optimization.

## Results

We recorded from five anesthetized owls of both sexes (four male and one female). Spatial tuning in free-field was measured with a high-density speaker array. The correlation structure [signal correlation (R*_sig_*), R*_NC_*], and spiking synchrony was assessed for pairs of simultaneously recorded neurons in each region. Correlation coefficients are reported as mean and standard deviation. All statistical tests performed are compiled in Table 1.

### Tuning properties of nearby units in OT

Single units recorded from OT (19 recording sites, *n* = 93 units) displayed well-delimited SpRFs (Fig. 2*A*), consistent with previous reports (Knudsen, 1982, 1984; Knudsen and Knudsen, 1983). Signal correlation analysis was used to assess the similarity of tuning properties for each pair of OT neurons in a recording site. Neighboring OT cells from the same recording site displayed similar tuning for azimuth (R*_sig_* = 0.61 ± 0.39, *n* = 193 pairs); an example of one recording site is presented in Figure 2*C*. The correlated tuning of neighboring cells in OT is consistent with the topographic representation of space, where nearby cells are tuned to nearby locations. Due to the topographic organization of spatial tuning in OT, the R*_sig_* is expected to vary with distance between recording sites (Knudsen, 1982). to adequately quantify the signal correlation across recording sites in OT, a systematic recording of distant regions of OT would be necessary. Similar recordings have previously been performed (Knudsen, 1982) and were deemed beyond the scope of this study. Thus, signal correlation analysis across sites in OT was also judged beyond the current goal. Based on previous descriptions of OT, a dataset consisting of a systematic sampling would yield low signal correlations across sites.

### Tunings properties in Field L

**Figure 3.**
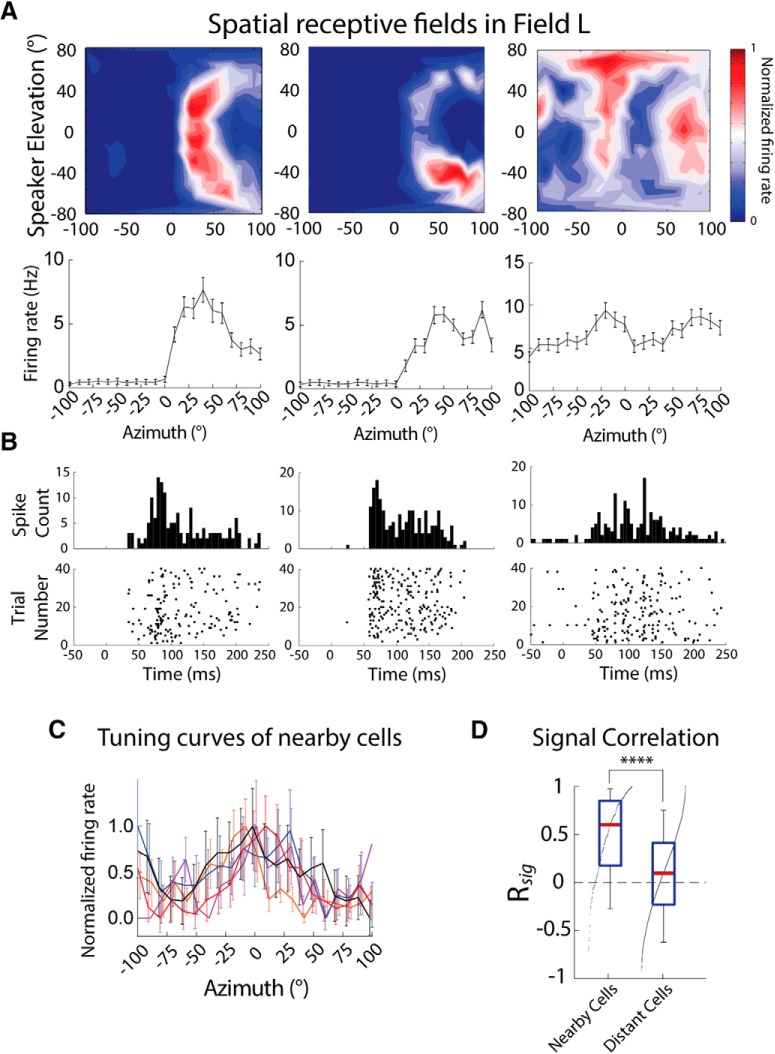
Azimuth tuning in Field L. ***A***, Example SpRFs (top) and azimuth tuning curves (bottom) of Field L neurons from different recording sites. ***B***, Peristimulus time histogram (PSTH; top) and rasters (bottom) for the spiking activity of the neurons in ***A***for sounds from the speakers eliciting the maximal response. ***C***, Example azimuth tuning curves of neurons recorded from the same site (different neurons from ***A***, ***B***). Firing rates are normalized to facilitate comparison. Tuning curves represent mean ± SEM, 20–40 repetitions. ***D***, R*_sig_* for azimuth tuning of nearby cells (left) and cells from different recording sites (right). Box plots show median (red line), interquartile range (blue), and 5% and 95% quantiles (whiskers). Black dots indicate the sorted distribution of data points. Asterisks indicate statistical significance (****p < 0.0001; two-tailed Mann–Whitney *U* test).

Azimuth tuning in Field L (22 recording sites, *n* = 116 units) was estimated by averaging SpRFs measured in free-field (Fig. 3*A*, top) across elevations (Fig. 3*A*, bottom). Tuning was less sharp than in OT as previously reported (Cohen and Knudsen, 1998). The preferred azimuth did not vary systematically along electrode tracks and between recording sites, consistent with previous reports that Field L is nontopographically organized with respect to spatial tuning (Cohen and Knudsen, 1998). Field L neurons from the same recording site displayed similar tuning (R*_sig_* = 0.50 ± 0.45, *n* = 300 pairs; Fig. 3*C*,*D*, left), consistent with a clustered distribution previously reported (Cohen and Knudsen, 1998).

Additionally, signal correlation was calculated for pairs of Field L neurons, across recording sites. R*_sig_* in pairs of cells recorded from different recording sites was lower than in pairs from the same recording site (0.10 ± 0.46, *n* = 4068 pairs; *p* < 0.0001, Mann-Whitney; Fig. 3*D*, right). The higher R*_sig_* in nearby cells than in cells across recording sites indicates that the tuning is more similar in neighboring cells than across the population. This result further supports the reports that clusters of similarly tuned cells are randomly distributed across Field L (Cohen and Knudsen, 1998).

### Tunings properties in AAr

**Figure 4.**
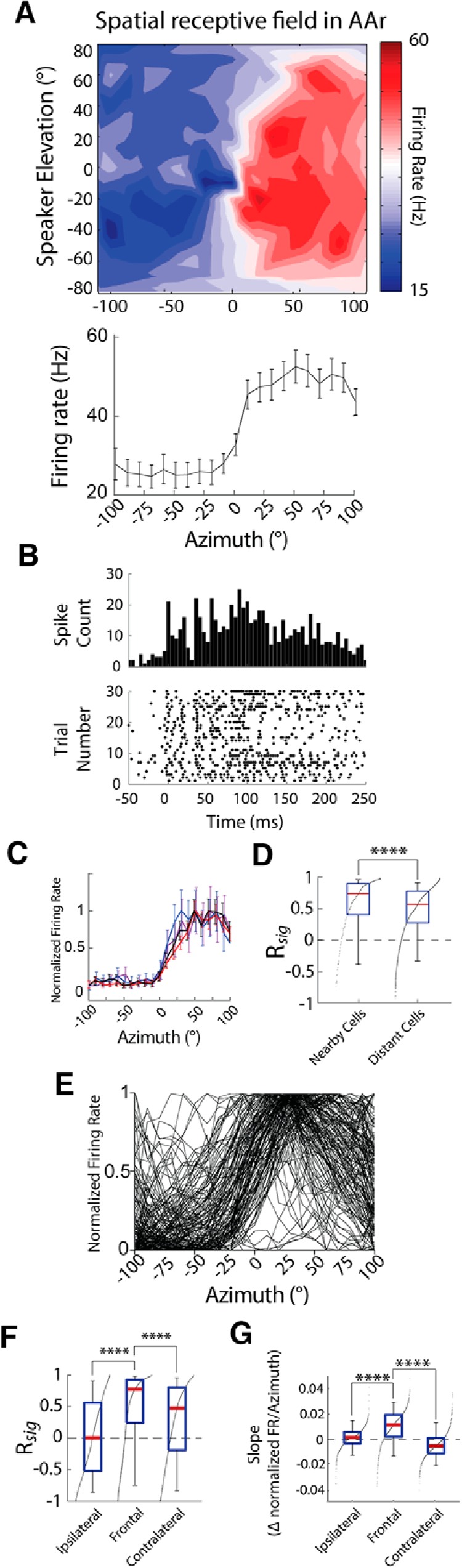
Spatial tuning in AAr. ***A***, Example SpRF (top) and azimuth tuning curve (bottom). ***B***, Peristimulus time histogram (PSTH; top) and rasters (bottom) for the spiking activity of the neuron in ***A***, stimulated by sound from the speaker that elicited the maximal response (20° azimuth and −20° elevation). ***C***, Example azimuth tuning curves of neurons recorded from the same site. Curves show normalized firing (mean ± SEM, 20–40 repetitions). ***D***, Signal correlation for azimuth tuning of nearby cells (left) and cells from different recording sites (right). ***E***, Overlaid azimuth tuning curves of all neurons in the AAr dataset. ***F***, Signal correlation within ipsilateral, frontal, and contralateral azimuth subregions of distant cells. ***G***, Steepness (slope) of azimuth tuning curves within ipsilateral, frontal, and contralateral space. Box plots in ***D***, ***F***, ***G***show median (red line), interquartile range (blue), and 5% and 95% quantiles (whiskers). Black dots indicate the sorted distribution of data points. ****p < 0.0001. ***D***, Two-tailed Mann–Whitney *U* test; ***F***, ***G***, Kruskal-Wallis *H* test with Dunn’s multiple comparisons correction.

AAr neurons (34 recording sites, *n* = 140 units) showed characteristic azimuth tuning shapes (Fig. 4*A*), in agreement with previous reports (Cohen and Knudsen, 1995; Vonderschen and Wagner, 2009, 2012). Tuning curves displayed a transition from lower to higher firing rate across the midline, with lower responses to sounds in the ipsilateral space (Fig. 4*A*,*C*). Interestingly, this shape is evocative of hemispheric responses proposed by the two-channel rate-code theory for sound localization in mammals (Békésy, 1930; van Bergeijk, 1962; McAlpine et al., 2001; Grothe et al., 2010). Consistently, R*_sig_* was high for azimuth tuning (0.61 ± 0.45, *n* = 252 pairs; Fig. 4*C*,*D*, left).

Unlike in Field L, similarity in azimuth tuning was also observed across AAr recording sites (R*_sig_* = 0.52 ± 0.40, *n* = 5874 pairs; Fig. 4*D*, right, *E*), with maximal correlation of tuning curve shapes in the front (Fig. 4*F*). To quantify this observation, we compared signal correlation in the front (±40°), contralateral (+50° to +100°), and ipsilateral (−100° to −50°) portions of azimuth tuning curves separately. R*_sig_* was significantly higher in frontal space (R*_sig_* = 0.57 ± 0.62) than the full azimuth range, ipsilateral (R*_sig_* = 0.03 ± 0.65), and contralateral space (R*_sig_* = 0.33 ± 0.65; all comparisons *p* < 0.0001, Kruskal-Wallis). Together, these data show higher R*_sig_* in AAr with particularly high R*_sig_* in the frontal space portion, across AAr neurons (Fig. 4*D*, right, *F*).

To characterize the information contained in AAr’s tuning curves, we measured the slope of these curves (Paradiso, 1988; Seung and Sompolinsky, 1993). The region that was most similar for azimuth tuning, i.e., the front, also harbored the steepest slopes (frontal: 0.0098 ± 0.0131; ipsilateral: 0.0009 ± 0.0087; contralateral: −0.0050 ± 0.0105 firing rate change/degree azimuth; all comparisons *p* < 0.0001, Kruskal-Wallis; Fig. 4*G*). This finding demonstrates that not only is the frontal portion of space most similar across the whole population in AAr, but that this region may be the most informative about the stimulus location, which has also been proposed to be important for a rate code (McAlpine et al., 2001; Grothe et al., 2010).

Taken together, these results show uniform spatial tuning in AAr, with responses increasing from the ipsilateral to the contralateral side across the front. Additionally, tuning curves across the population were most correlated in the frontal portion of space, which was also the most informative (steeper slope) about auditory space.

### Signal correlation across structures

**Figure 5.**
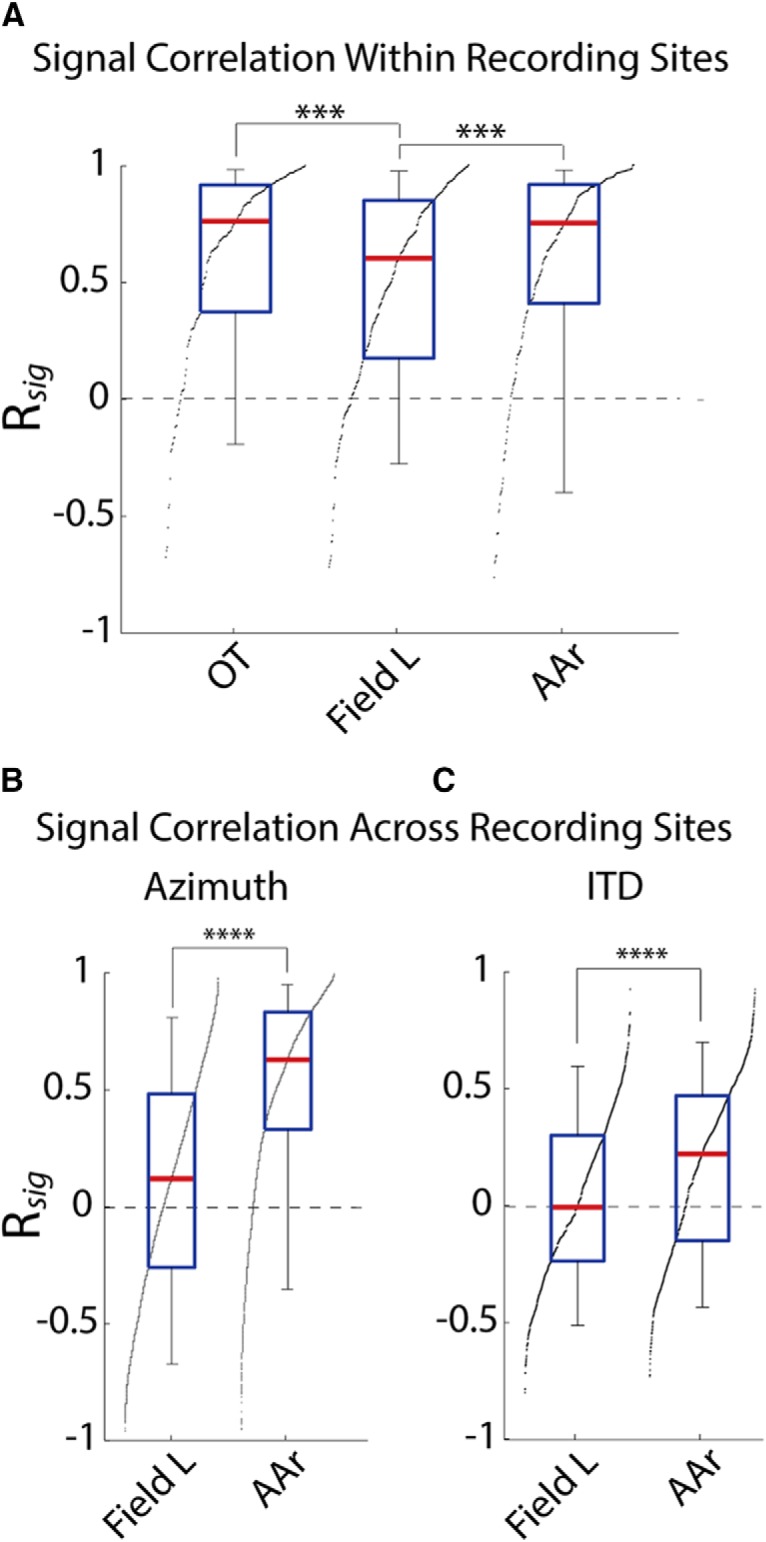
Comparison of signal correlation across brain regions. ***A***, Signal correlation in nearby cells for azimuth tuning. ***B***, ***C***, Signal correlation across recording sites, for azimuth (***B***) and ITD (***C***) tuning. The significantly stronger signal correlation across distant cells in AAr corroborates a more homogeneous tuning than in Field L. Box plots show median (red line), interquartile range (blue), and 5% and 95% quantiles (whiskers). Black dots indicate the sorted distribution of raw values. ***p < 0.001, ****p < 0.0001. ***A***, Kruskal-Wallis *H* test with Dunn’s multiple comparisons correction; ***B***, ***C***, two-tailed Mann–Whitney *U* test.

In all regions tested, signal correlation was high in neighboring cells. This was anticipated, given the topographic organization in OT and the previously reported clusters of similarly tuned neurons in the forebrain (Cohen and Knudsen, 1995, 1998). Interestingly, signal correlation of nearby neurons in AAr was significantly higher than in Field L (*p* = 0.0007, Kruskal-Wallis; Fig. 5*A*). Moreover, signal correlation across recording sites was also significantly higher in AAr than in Field L (*p* < 0.0001, Mann-Whitney; Fig. 5*B*). This suggests a transformation from a cluster organization in Field L into a homogeneously tuned population in AAr. Recordings were targeted throughout the anatomic extent of AAr of multiple subjects. Because AAr does not display a topographic representation of auditory space (Cohen and Knudsen, 1995), the higher signal correlation across recording sites is unlikely due to over-sampling a particular region of AAr. Instead, these results indicate AAr neurons are more similarly tuned regardless of proximity. While the homogeneous tuning across AAr is not completely unexpected, as previous reports have noted the characteristic tuning shape and have described how it may form (Vonderschen and Wagner, 2012), it highlights the effect the correlation structure should have on coding. To further confirm the transformation from Field L to AAr, we also compared the similarity of ITD tuning obtained with dichotic stimulation (AAr: 13 recording sites, 44 neurons; Field L: 10 recording sites, 46 neurons). Consistently, the ITD tuning was also significantly more similar across recording sites in AAr than Field L (AAr: R*_sig_* = 0.18 ± 0.39, *n* = 493 pairs; Field L: R*_sig_* = 0.03 ± 0.36, *n* = 469 pairs; *p* < 0.0001, Kruskal-Wallis), further demonstrating neurons across AAr display similar tuning (Fig. 5*C*). Overall, these results show a unique, homogeneous, organization of spatial tuning in AAr, which is different from both Field L (clustered) and OT (topographic).

### R*_NC_* across structures

**Figure 6.**
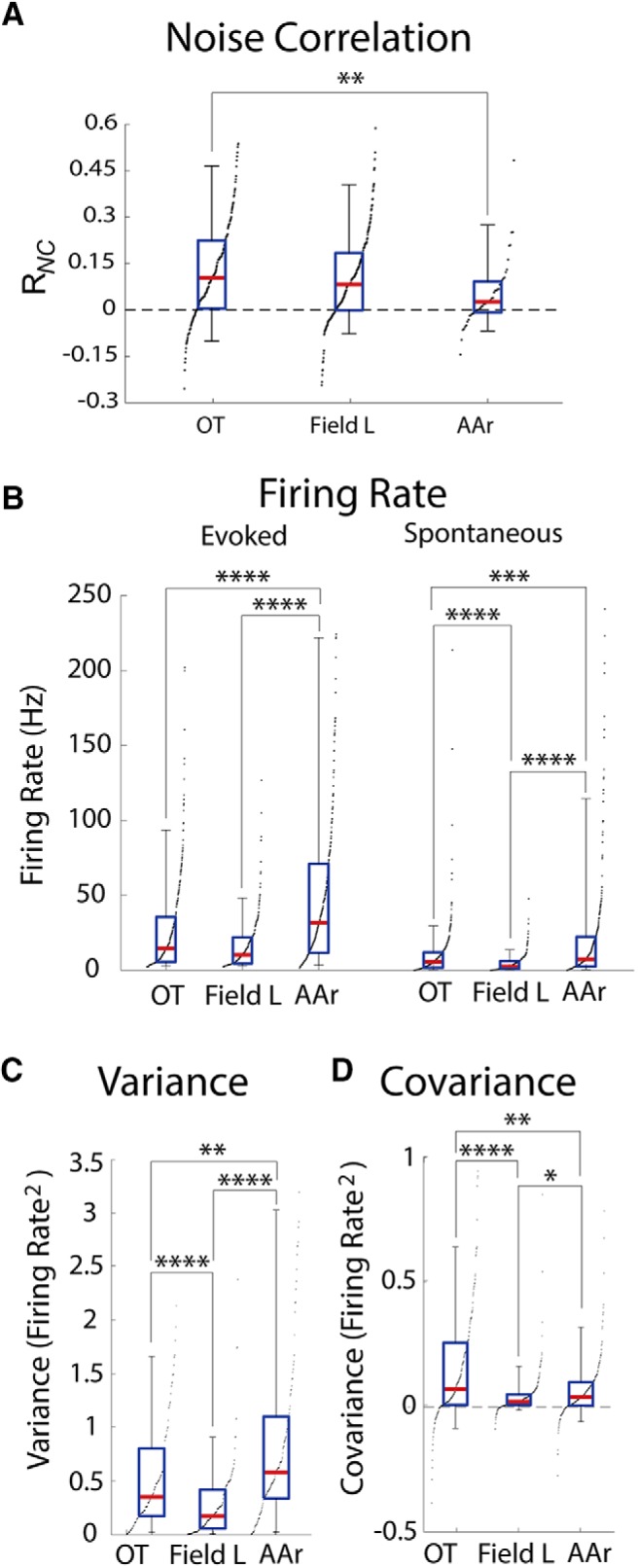
Comparison of R*_NC_*s. ***A***, R*_NC_*s in OT, Field L, and AAr. ***B***, Average firing rates of OT, Field L, and AAr cells during sound presentation (left) and spontaneous firing (right). ***C***, ***D***, Average variance (***C***) and covariance (***D***) in OT, Field L, and AAr neurons. Box plots represent median (red line), interquartile range (blue), and 5% and 95% quantiles (whiskers). Black dots indicate the sorted distribution of raw values. *p < 0.05, **p < 0.01, ***p < 0.001, ****p < 0.0001; Kruskal-Wallis *H* test with Dunn’s multiple comparisons correction.

R*_NC_* in simultaneously recorded neurons was assessed in all three structures by measuring the covariability of spike numbers elicited by repeated trials of frozen broadband noise. R*_NC_*s in OT were near values reported in studies in the visual cortex (Smith and Kohn, 2008; Ponce-Alvarez et al., 2013), somatosensory cortex (Okun et al., 2015), the songbird auditory forebrain (Jeanne et al., 2013), the mammalian A1 (Downer et al., 2015), and used in computational models (Cohen and Kohn, 2011; Kohn et al., 2016; R*_NC_* = 0.13 ± 0.17, *n* = 168 pairs; Kruskal-Wallis; Fig. 6*A*). R*_NC_*s in AAr were significantly smaller than in OT (R*_NC_* = 0.06 ± 0.13, *n* = 48 pairs; OT vs AAr: *p* = 0.0088, Kruskal-Wallis; Fig. 6*A*). R*_NC_*s in Field L, on the other hand, were intermediate between OT and AAr, and not significantly different from either (Field L: R*_NC_* = 0.11 ± 0.16, *n* = 225 pairs; Field L vs OT: *p* = 0.54, Field L vs AAr: *p* = 0.08; Kruskal-Wallis; Fig. 6*A*). These results show lower R*_NC_*s in AAr than in the midbrain.

Next, we tested potential causes of low R*_NC_*s in the forebrain. Mathematically, R*_NC_*s are independent from firing rate. However, biological mechanisms such as the spiking threshold nonlinearity, may induce lower R*_NC_*s at low firing rates (Cohen and Kohn, 2011). Additionally, an *in vitro* study has demonstrated that R*_NC_*s are higher for neurons with higher firing rates (de la Rocha et al., 2007). To test if firing rates could explain the lower R*_NC_*s in the forebrain, the spontaneous and evoked firing rates were compared across structures (Fig. 6*B*). AAr’s firing rate was significantly higher than both Field L and OT and Field L’s spontaneous firing rate was significantly lower than in the other structures (spontaneous firing rate: OT = 9.83 ± 17.13; Field L = 4.45 ± 5.74; AAr = 23.54 ± 48.47; evoked firing rate: OT = 29.11 ± 43.75; Field L = 16.24 ± 17.30; AAr = 57.87 ± 80.27 spikes/s; spontaneous OT vs AAr: *p* = 0.0003; evoked OT vs Field L: *p* = 0.059; all other comparisons; *p* < 0.0001, Kruskal-Wallis; Fig. 6*B*). While the comparatively low R*_NC_*s observed in Field L could be attributed to a low firing rate, the higher firing rate in AAr cannot explain its significantly reduced R*_NC_*.

Because R*_NC_* is inversely related to the standard deviation of individual responses and directly related to covariance, low R*_NC_* can result from increased variance, decreased covariance, or both. While single-cell mechanisms may drive the variance of individual cell responses, the covariance may reflect properties of network architecture such as shared inputs. We therefore examined response variance and covariance across the dataset. Interestingly, individual AAr neurons displayed significantly higher variance, compared with OT and Field L [OT: 0.66 ± 1.49; Field L: 0.29 ± 0.35; AAr: 1.08 ± 1.69 firing rate (spikes/s)^2^; both comparisons; *p* < 0.0001, Kruskal-Wallis; Fig. 6*C*] and significantly lower covariance than OT [OT: 0.17 ± 0.30; Field L: 0.04 ± 0.07; AAr: 0.08 ± 0.19 firing rate (spikes/s)^2^; OT vs AAr: *p* = 0.0095, Field L vs AAr: *p* = 0.045 Kruskal-Wallis; Fig. 6*D*]. Therefore, the low R*_NC_* in AAr results from both increased variability of individual neurons’ responses and reduced covariability, suggesting that single-cell and network mechanisms impact R*_NC_*s.

### Spike synchrony across structures

**Figure 7.**
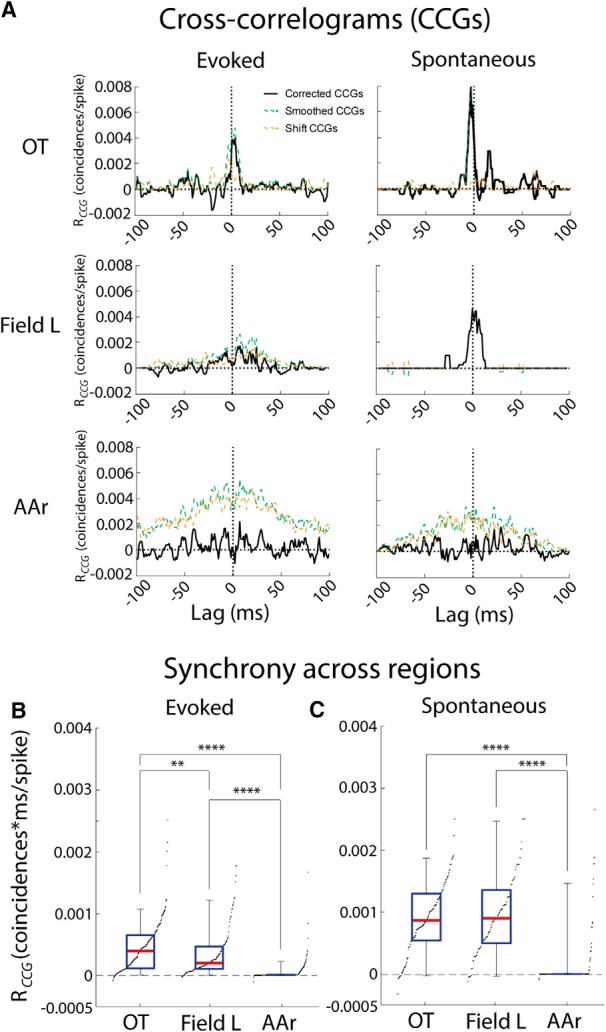
Spike-time synchrony in OT, Field L, and AAr. ***A***, Example CCGs for pairs of evoked (left) and spontaneous (right) responses in OT (top), Field L (middle), and AAr (bottom). The corrected CCGs for individual pairs of neurons (solid black) are overlaid to the smoothed CCG (dashed green) and shifted CCG (dashed orange). The plots for left and right are from the same pair. ***B***, ***C***, Statistical comparison of synchrony across brain regions for evoked (***B***) and spontaneous (***C***) spikes. Box plots represent median (red line), interquartile range (blue), and 5% and 95% quantiles (whiskers). Black dots indicate the sorted data; **p < 0.01, ****p < 0.0001; Kruskal-Wallis *H* test with Dunn’s multiple comparisons correction.

To achieve further insight into mechanisms underlying the different correlation structures observed in midbrain and forebrain, spike synchrony was computed for all pairs of simultaneously recorded neurons. Spike synchrony has been linked to shared connections between neurons (Atencio and Schreiner, 2013; Miller et al., 2014; Atencio et al., 2016; Sabri et al., 2016; Schwab et al., 2017; Yaeger and Trussell, 2016). Thus, lack of shared connections between neurons could underlie the low R*_NC_* in the forebrain. To assess the synchrony of spikes in pairs of neurons, CCGs were calculated from simultaneously recorded spike trains. Synchrony was then quantified by taking the integral of the peak at 0-lag. The peak’s boundaries were set at half maximum response. Synchrony was significantly different for all three regions with OT displaying the highest synchrony and AAr displaying the lowest [peak CCG integral: OT = 0.00052 ± 0.00054, *n* = 116 pairs; Field L = 0.00025 ± 0.00038, *n* = 102 pairs; AAr = 0.000032 ± 0.00019 coincidences*ms/spike, *n* = 219 pairs; OT vs FL: *p* = 0.0067, OT vs AAr: *p* < 0.0001, Field L vs AAr: Kruskal-Wallis; Fig. 7*A*, left, *B*]. Synchrony during spontaneous activity was similar in Field L and OT and both were larger than in AAr (OT = 0.00091 ± 0.00058, *n* = 136; peak CCG: Field L = 0.00100 ± 0.00062, *n* = 83; AAr = 0.00016 ± 0.0006 coincidences*ms/spike, *n* = 207; OT vs Field L: *p* > 0.9999, OT vs AAr: *p* < 0.0001, Field L vs AAr: *p* < 0.0001, Kruskal-Wallis; Fig. 7*A*, right, *C*). Synchrony was lower during evoked than spontaneous spiking for both OT and Field L (spontaneous vs evoked: OT, *p* = 0.03; Field L, *p* < 0.0001; Kruskal-Wallis). Decreased synchrony from spontaneous to evoked responses has been observed in other brain regions (Tsodyks et al., 1999; Kohn and Smith, 2005). AAr did not display this relationship, likely due to a floor effect, as synchrony during spontaneous spiking was already close to zero. Therefore, nearby neurons in AAr, although having similar tuning, fired spikes in a remarkably independent fashion, suggesting lack of shared input. Similar to R*_NC_*s, synchrony measured with CCGs is also influenced by firing rate. In particular, higher firing rates increase the number of coincidences and elevate the magnitude of the center peak of the CCG, artificially increasing synchrony, even with normalization (de la Rocha et al., 2007; Smith and Kohn, 2008). As was shown in Figure 6*B*, AAr neurons displayed higher firing rates than OT and Field L. Therefore, as was the case for R*_NC_*s, the reduced synchrony in AAr cannot be explained by differences in firing rates.

### Effect of correlations on information decoding

**Figure 8.**
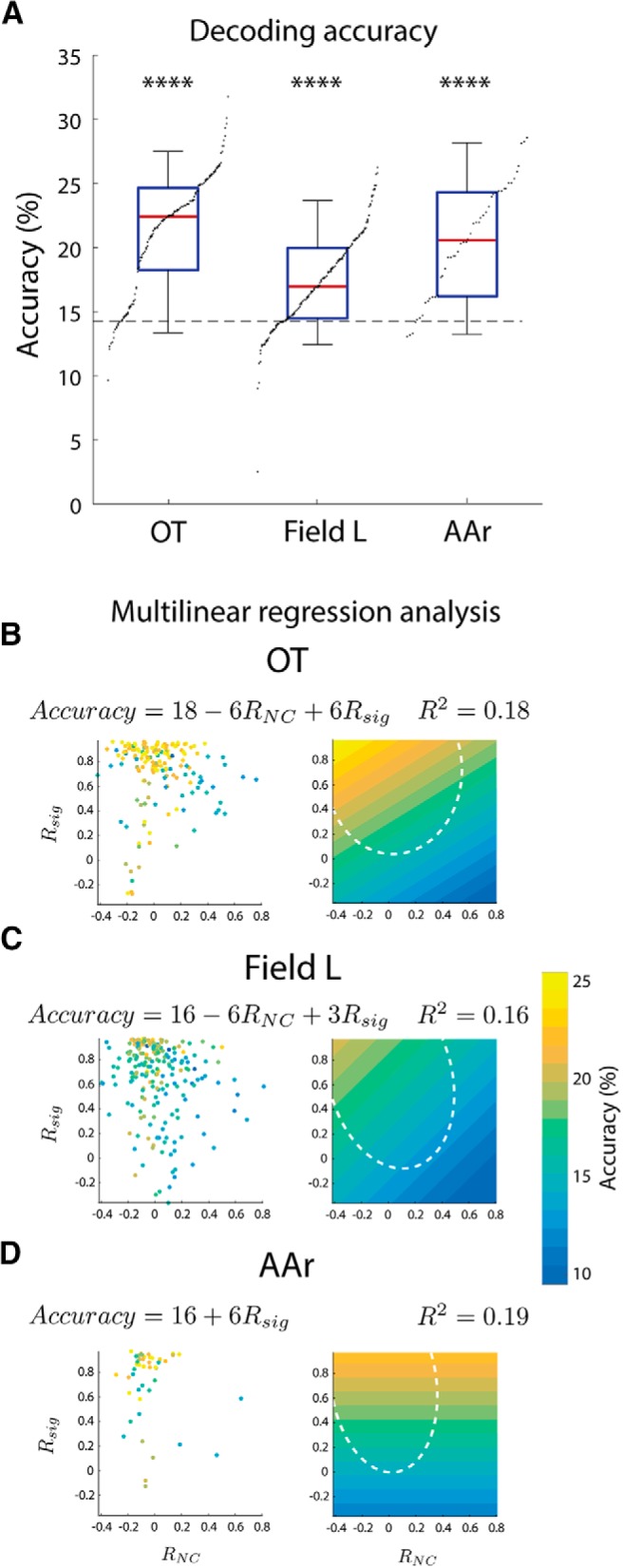
Decoding of ITD and azimuth from firing rate. ***A***, Decoding accuracy in pairs of simultaneously recorded units in OT, Field L, and AAr. Box plots represent median (red line), interquartile range (blue), and 5% and 95% quantiles (whiskers). Asterisks indicate better than chance level decoding of azimuth (dashed line: 14.92°). Dots are the sorted data points. ***B–D***, left, Decoder performance (colored points) plotted against signal (R*_sig_*) and R*_NC_* for each pair of neurons in OT (***B***), Field L (***C***), and AAr (***D***). Point color indicates level of accuracy (color bar on the right). Right, Linear fit of accuracy data as a function of signal and R*_NC_*. White dashed ellipsoids depict 95% range of signal and R*_NC_*s used for the linear fit, which avoided outliers. Fit functions and *R* ^2^ values are shown above each plot. Color bar matches all plots (****p < 0.0001; Wilcoxon signed-rank test).

Previous studies have shown dramatic effects of the correlation structure on the amount of information a neural system can encode (Averbeck and Lee, 2006; Averbeck et al., 2006). In particular, high R*_NC_*s may limit the amount of information in a population with high signal correlations. Additionally, R*_NC_* limits information in rate-code schemes (Sompolinsky et al., 2001; Zohar et al., 2013). To assess whether the different correlation structures in the midbrain and forebrain may have consequences on coding we estimated the accuracy of simultaneously recorded pairs of neurons in encoding azimuth, using linear discriminant analysis (Materials and Methods). This decoder considers the spike counts while attempting to determine the optimal decoding strategy. This strategy allows the decoder to use the biological R*_NC_*s. However, limiting the decoding estimate to pairs of neurons reduces information. Additionally, most simultaneously recorded neurons displayed similar tuning, suggesting their responses carry overlapping information. Both of these factors would inherently make the decoder perform worse. Impressively, even with these limitations, the classifier identified the azimuth above chance levels in all three regions (OT: 21.21 ± 4.59%; Field L: 17.23 ± 3.57%; AAr: 20.53 ± 4.37%; *p* < 0.0001, Wilcoxon signed-rank; Fig. 8*A*).

Decoding performance can be dictated by numerous factors (e.g., variance, tuning shape, steepness of curves), including the correlation structure. For example, high signal and R*_NC_*s together can impair performance (Averbeck et al., 2006). Multilinear regression analysis was used to quantify how predictive the correlation structure was of the decoder’s accuracy, an assessment of the effect of correlation structure on the readout of the population. Signal and R*_NC_*s could explain 18% and 16% of the variance of the decoding performance for OT and Field L, respectively (Fig. 8*B*,*C*). This suggests that noise and signal correlations have a significant effect on decoding accuracy. In particular, good classification was associated with stronger signal and weaker R*_NC_*s, reflecting that high R*_NC_* limits information carried in the firing rate of populations of neurons with similar tuning. Interestingly, this trend was not observed in AAr. Here 19% of the variance of the decoding performance could be explained by signal correlation alone (Fig. 8*D*). Including R*_NC_* did not increase the predictive power of the model (*R* ^2^ = 0.19 with both signal and R*_NC_*s), likely due to the narrow range of R*_NC_*s observed in AAr with mean close to zero. This suggests that while R*_NC_*s were detrimental for OT and Field L, they were of no consequence in AAr. Thus, the low R*_NC_*s in AAr may be beneficial for coding by restricting the information-limiting effect of R*_NC_*s.

**Figure 9.**
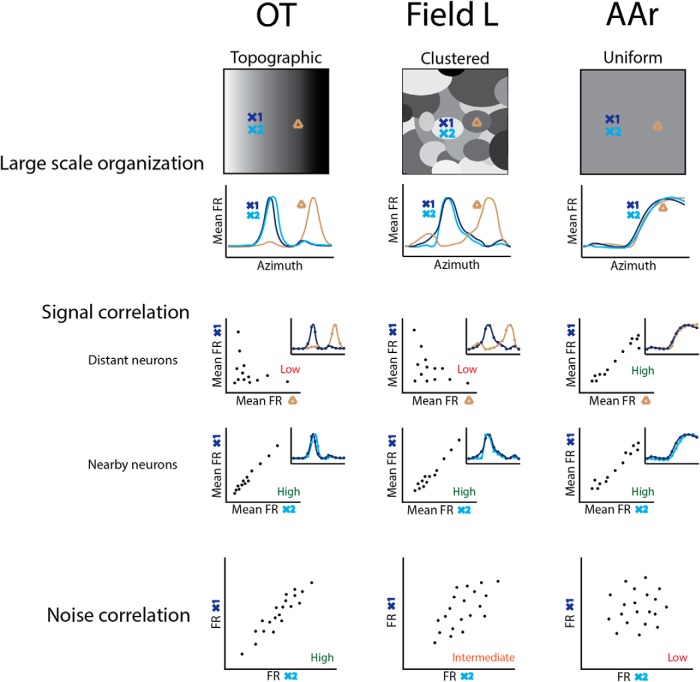
Summary of findings. Top, Large-scale spatial tuning organization of each region (above) and corresponding schematic tuning curves at recording locations (below) denoted by crosses (X1 and X2 represent nearby sites) and triangles representing a distant site. OT displays a topographic organization of spatial tuning, while Field L is organized in clusters. AAr displays uniform tuning. Middle, Signal correlation for distant (above) and nearby (below) neurons. Tuning curves of distant sites is different in OT (extrapolated from previous descriptions) and Field L but similar in AAr (insets). On the other hand, tuning curves of nearby neurons are similar in all three structures. Scatter plots represent firing rates (FR) of pairs of cells across azimuth plotted against one another, used to calculate signal correlation. Tuning curves are shown in the insets. Bottom, Schematic scatter plots representing the correlated FR variability of nearby cells in OT, intermediate level of FR variability in Field L, and uncorrelated FR variability in AAr.

In sum, these results demonstrate a different correlation structure in the midbrain and forebrain (Fig. 9). Nearby neurons in OT were more similarly tuned than in the forebrain, with strongly correlated tuning, firing rate variability, and spike timing. In the forebrain, on the other hand, Field L contained clusters of similarly tuned neurons distributed randomly. Whereas AAr neurons were more uniformly tuned, with their firing rate increasing from ipsilateral to contralateral space across the front, but displaying uncorrelated variability and timing. While the correlation structure in the midbrain can be explained by shared inputs of nearby cells in a topographic representation, the forebrain neurons responded more independently over time, suggesting decreased shared inputs. Our decoding approach showed that this unique correlation structure in AAr is advantageous under the two-channel rate-code scheme of sound location (van Bergeijk, 1962; McAlpine et al., 2001; Grothe et al., 2010).

## Discussion

We found distinct correlation structure in the owl’s auditory midbrain and forebrain, characterized primarily by significantly lower R*_NC_* in the forebrain. In particular, a unique correlation structure emerges in AAr, with high signal correlation between nearby and also distant cells, but low R*_NC_*. Thus, the tuning of AAr neurons is uniform across the population while variability is decorrelated. A decoder model shows this correlation structure can limit the effect of R*_NC_* on information. Interestingly, the tuning shape in AAr is reminiscent of the two-channel rate code for sound localization (van Bergeijk, 1962; McAlpine et al., 2001; Grothe et al., 2010). Thus, the AAr correlation structure may be beneficial for reliable rate coding of auditory space in the forebrain.

### Emergence of uniform tuning in the forebrain

The high signal correlation in nearby neurons in OT and Field L corroborates reports of similar tuning of nearby cells in both structures. While the map of auditory space in OT determines that nearby cells are tuned to nearby positions in space (Knudsen, 1982), previous studies have reported clusters of similarly tuned neurons in Field L with no topographic organization which have been compared with cortical columns (Cohen and Knudsen, 1995, 1998). The low signal correlation across recording sites in Field L supports the cluster hypothesis. On the other hand, AAr showed strong signal correlations within and across recording sites, indicating that the tuning is homogeneous across the population. These results provide further evidence supporting previous descriptions of tuning in OT and Field L, while highlighting AAr as a more homogeneous population.

### Emergence of uncorrelated firing in the forebrain

Response covariability of nearby neurons in OT, Field L, and AAr differed. Variability in OT was correlated and spikes were synchronous, suggesting neighboring OT cells share inputs (Smith and Kohn, 2008; Smith and Sommer, 2013; Downer et al., 2015).

Because low firing rates have been associated to lower R*_NC_*s (de la Rocha et al., 2007; Cohen and Kohn, 2011), it is possible that the lower R*_NC_* in Field L results from an effect of the spiking nonlinearity on correlated but weak subthreshold inputs. Field L neurons also displayed synchronous spontaneous spiking; suggesting these neurons may receive shared thalamic input. However, synchrony was reduced in evoked spikes. Increased decorrelation in stimulus-driven responses has been reported (Tsodyks et al., 1999; Kohn and Smith, 2005).

Interestingly, AAr neurons displayed high firing rate, while correlated variability, R*_NC_* and synchrony, were close to zero. This suggests that mechanisms to dampen R*_NC_* may exist in AAr. Both network and cellular mechanisms can reduce R*_NC_* (Shadlen and Newsome, 1998; Azouz and Gray, 1999; Wiechert et al., 2010; Ribrault et al., 2011; Tetzlaff et al., 2012; Grytskyy et al., 2013; Herrero et al., 2013; Goris et al., 2014; Chelaru and Dragoi, 2016; von Trapp et al., 2016). The analysis of variance and covariance can provide insight into what mechanisms may be in place. In AAr, the covariance was low while the variance of individual neuron responses was high, both of which would result in low R*_NC_*s. Thus, mechanisms influencing both the variability of responses of individual neurons and the joint variability of pairs of neurons may be in place. For example, recurrent inhibition (Wiechert et al., 2010; Tetzlaff et al., 2012; Grytskyy et al., 2013; Chelaru and Dragoi, 2016) and higher variability of synaptic release (Ribrault et al., 2011) have been demonstrated to reduce R*_NC_*s and are viable mechanisms for AAr. Recurrent inhibition could be implemented in AAr through reciprocal connections across hemispheres, or feedback from one of AAr’s downstream targets (Knudsen et al., 1995). Another potential mechanism may be lack of shared inputs, which are suggested by the low synchrony in AAr. Further investigation of the circuit and cellular properties of AAr neurons is necessary to fully understand the mechanisms underlying uncorrelated firing in AAr.

Recordings were performed on anesthetized owls. This eliminated the effect of changes in alertness on R*_NC_*s (Cohen and Maunsell, 2009; Herrero et al., 2013). However, anesthesia has been shown to differently affect forebrain and midbrain responses. Specifically, the spectrotemporal tuning of midbrain responses remain largely unaffected by anesthesia while it broadens in the forebrain (Capsius and Leppelsack, 1996; Alkire and Miller, 2005; Schumacher et al., 2011; Karino et al., 2016). Thus, it is possible the different R*_NC_*s in midbrain and forebrain could be generated through a differential effect of anesthesia. However, previous work has reported anesthesia increases R*_NC_*s in the forebrain by generating large “up and down” states of activity and quiescent periods (Ecker et al., 2014). While our recordings did not display such activity patterns, this effect would be inconsistent with the reduced R*_NC_* we observed in the forebrain.

### Correlation structure of the forebrain and implications for coding

Our results demonstrate that OT, Field L, and AAr have strikingly different correlation structures. Because correlated firing determines information, these differences may carry important implications for coding of auditory space in the midbrain and forebrain.

Tuning curves of AAr neurons showed the strongest signal correlation in the front, with a sharp transition from low firing rates in ipsilateral space to higher in contralateral space. The transition across the midline is reminiscent of the two-channel rate code. This hypothetical code relies on the average firing rate of two hemispheric populations and discriminates with greatest precision stimulus located in the front, the region of steepest slope (Békésy, 1930; van Bergeijk, 1962; Grothe et al., 2010; Razak, 2011; Lee and Groh, 2014; Briley et al., 2016). Correlated activity can greatly affect average responses of a uniformly tuned population (Zohary et al., 1994; Shadlen and Newsome, 1998; Zohar et al., 2013; Kohn et al., 2016), hindering the system’s discriminability. Therefore, the reduced R*_NC_*s in AAr are beneficial for a rate-code system. Additionally, we showed that R*_NC_*s are detrimental for decoding of sound source azimuthal location, and that the low R*_NC_* in AAr limits this effect. Taken together, these results demonstrate that a rate-code scheme would benefit from AAr’s correlation structure.

The presence or absence of maps of auditory space in the brain has mystified researchers, leading to different theories of how sound direction is represented (Békésy, 1930; Jeffress, 1948; McAlpine et al., 2001; Schnupp and Carr, 2009). Whereas maps have been associated to place code, a rate code has been suggested to explain the lack of it (Konishi, 2003; Schnupp and Carr, 2009; Grothe et al., 2010). Responses in the forebrain are reminiscent of the two-channel rate code proposed for rodents (McAlpine et al., 2001; Grothe et al., 2010) and humans (Briley et al., 2013; Derey et al., 2016; Dykstra et al., 2016; McLaughlin et al., 2016). Thus, our findings may generalize to other species. Our results provide support to the notion that auditory forebrain regions involved in sound localization, which do not exhibit a map, display a correlation structure favorable to a rate code.

### Concluding remarks

The comparative analysis throughout the owl’s sound localization system showed differences between the correlation structure in midbrain and forebrain. These findings permit a glimpse into how auditory space may be encoded in the forebrain, where a population with homogenous tuning but uncorrelated variability emerges. This correlation structure is beneficial under a rate-code framework. Additional evidence for the existence of the rate code and mapping the anatomic connections will be necessary to further test this hypothesis. These findings can be applicable to other species, as the topographic representation of auditory space in the superior colliculus (Cynader and Berman, 1972; Gordon, 1973; Knudsen, 1982; Palmer and King, 1982; Middlebrooks and Knudsen, 1984) and nontopographic in the forebrain exist in all species studied (Eisenman, 1974; Benson et al., 1981; Middlebrooks and Pettigrew, 1981; Imig et al., 1990; Rajan et al., 1990; Carr and Christensen-Dalsgaard, 2015; Razak et al., 2015; McLaughlin et al., 2016), and the two-channel rate code has been proposed for mammalian species (McAlpine et al., 2001; Derey et al., 2016).

**Table 1.** Summary of statistics

Source figure	Test	Sample size (*n*)	Test statistics	*p*	Power α = 0.05
3*D*	Mann-Whitney *U*	Within, 300; across, 4068	*U* = 301,457	<0.0001	1
4*D*	Mann-Whitney *U*	Within, 252; across, 5874	*U* = 559,821	<0.0001	0.99
4*F*	Kruskal-Wallis *H*	Full, 5875; ipsilateral, 5791; frontal, 5874; contralateral, 5874	*H* = 23,413	<0.0001 for all	1
4*G*	Kruskal-Wallis *H*	Ipsilateral, 700; frontal, 700; contralateral, 700	*H* = 533.1	<0.0001 for all	1
5*A*	Kruskal-Wallis *H*	OT, 193; Field L, 300; AAr, 252	*H* = 19.3	OT vs Field L = 0.0006 Field L vs AAr = 0.0007	1
5*B*	Mann-Whitney *U*	Field L, 4068; AAr, 5974	*U* = 5,671,377	<0.0001	1
5*C*	Mann-Whitney *U*	Field L, 469; AAr, 493	*U* = 361,864	<0.0001	0.99
6*A*	Kruskal-Wallis *H*	OT, 168; Field L, 225; AAr, 48	*H* = 8.93	OT vs AAr = 0.0088	0.985
6*B*	Kruskal-Wallis *H*	OT, 332; Field L, 259; AAr, 302	*H* = 95.6	OT vs Field L < 0.0001 OT vs AAr = 0.0003 Field L vs AAr < 0.0001	1
6*B*	Kruskal-Wallis *H*	OT, 359; Field L, 268; AAr, 323	*H* = 90.6	OT vs AAr < 0.0001 Field L vs AAr < 0.0001	1
6*C*	Kruskal-Wallis *H*	OT, 102; Field L, 121; AAr, 142	*H* = 63.7	OT vs FL < 0.0001; OT vs AAr = 0.0093 FL vs AAr < 0.0001	1
6*D*	Kruskal-Wallis *H*	OT, 232; Field L, 294; AAr, 235	*H* = 28.9	OT vs FL < 0.0001; OT vs AAr = 0.0049 FL vs AAr = 0.034	1
7*B*	Kruskal-Wallis *H*	OT, 116; Field L, 102; AAr, 219	*H* = 172.4	OT vs AAr < 0.0001 Field L vs AAr < 0.0001	1
7*C*	Kruskal-Wallis *H*	OT, 136; Field L, 83; AAr, 207	*H* = 184	OT vs FL = 0.0067 OT vs AAr < 0.0001; FL vs AAr < 0.0001	1
8*A*	Wilcoxon *T*	OT, 168	*T* = 14,196	<0.0001	1
8*A*	Wilcoxon *T*	Field L, 225	*T* = 25,425	<0.0001	1
8*A*	Wilcoxon *T*	AAr, 48	*T* = 1,176	<0.0001	1

Test statistics; *U* for Mann-Whitney, *H* for Kruskal-Wallis, and *T* for Wilcoxon.

## Synthesis

Reviewing Editor: Tatyana Sharpee, The Salk Institute for Biological Studies Decisions are customarily a result of the Reviewing Editor and the peer reviewers coming together and discussing their recommendations until a consensus is reached. When revisions are invited, a fact-based synthesis statement explaining their decision and outlining what is needed to prepare a revision will be listed below. The following reviewer(s) agreed to reveal their identity: Alan Palmer.

Thank you for doing the revisions. After the discussion with reviewers, it seems that only minor (but important) changes are needed. First, it would help to revise the abstract further, in particular the term correlation structure might not be clear. Second, the data in Fig. 5B is plotted wrong because it is not consistent with either the text or data in other figures. Third, y-axis labeling in Fig. 2B, 3B, and 4B should be trial number or equivalent. Fourth, please consider discussing results on the human auditory system included in the reviewer comments.

The authors have clearly done a considerable amount of work to revise the paper according to the previous reviewers' suggestions. That, combined with the fact that I am not an expert in microelectrode recordings or the types of analyses carried out by the authors means that I will try to focus mostly on major outstanding issues as well as offer suggestions on minor points.

First, in line with the previous reviewers, the abstract could still be clarified considerably, particularly regarding exactly what was measured or computed in examining “correlation strucutres”. While this might be second nature to the authors, many readers will not know what this means a priori. It is therefore worth being more explicit and less jargony, particularly in the abstract, about what a correlation structure is, how it's computed, and what measurements are made in order to do so. At present, precise definitions of signal and noise correlations doesn't come until deep in the methods section. I would strongly consider adding similar text to the introduction, and maybe even brief versions in the abstract, which will help the reader appreciate the motivation and outline of the study.

In the abstract: “In addition to the difference in tuning of adjacent neurons, the co-variability of responses (i.e. correlation structure) in topographic and non-topographic representations is expected to differ, as both are determined by the underlying circuitry.”

It is not immediately apparent why different circuits couldn't produce similar correlation structures (the work of Eve Marder, for example). I would consider deemphasizing this prediction and focus more on the fact that they turn out to be different. Also, as stated by previous reviewers, it would be worthwhile for the authors to be more explicit and less jargony in the abstract about exactly what they mean by correlation structure.

Other major issues:

I am confused by the y-axis labeling of the lower panels of Figs. 2B, 3B, and 4B. These seem to be dot rasters, and as such the y-axis should be labeled “trial number”, no?

Minor issues:

In the abstract: Consider omitting “For their critical relevance on information” from that sentence.

Line 25: “entire hearing range”

State the range.

Line 28: “Lesion studies have demonstrated that both pathways are capable of supporting orienting behavior towards sound.”

Wouldn't lesion studies allow one to conclude with more certainty that a certain area, in fact, does support a particular behavior?

Lines 31-33: analogue → analogous

Lines 34-36: “This non-topographic organization is also observed in the auditory cortex of all species studied.”

I would mention the species individually to give more weight to the argument, and also mention a few relevant human studies. For example:

McLaughlin et al. (2015) J Assoc Res Otolaryngol.

Dykstra et al. (2016) J Assoc Res Otolaryngol.

Lines 137-138: “The slope of the tuning curves within each of these regions was assessed computing the mean slope of linear regressions for a sliding window of 3 consecutive curve data points ...”

“... assessed by computing ...”

Lines 210-217: Consider omitting. It's a rehash of what's already in the introduction and methods sections.

Lines 226-227: “Thus signal correlation analysis across distant sites in OT was judged beyond the current goal. Based upon previous descriptions of OT, a data set consisting of a systematic sampling would yield low signal correlations across sites.”

Why not do the analysis given 19 recording sites and then compare within vs. across sites, as was done for Field L and AAr?

Lines 268-269: “Moreover, signal correlation across recording sites was also significantly higher in AAr than in Field L (p<0.0001, Mann-Whitney; Figure 5B).”

Is Fig. 5B correct? Shouldn't the values shown here be similar (if not identical) to those shown in Fig. 3D (for Field L) and Fig. 4D (for field AAr)? As it appears currently, the distributions are quite overlapping, and both seem Gaussian. If anything, the median differences would seem to suggest the reverse effect from what the authors describe. What the U-test might be indicating here is that the distributions are not the same, rather than the median value of one being larger than the median value of the other. Relatedly, for all statistical tests, the authors should report the test statistic (in this case, U). Finally, as far as I know, there is not such thing as a “Mann-Whitney U t-test” (line 676).

In the discussion, I would like to see a bit more about how the results might relate to how space is represented in the human auditory system.
